# 1640. Acyclovir use in infants and children (0-18 years) in Pediatric hospitals in Australia and New Zealand for suspected herpes simplex virus infection

**DOI:** 10.1093/ofid/ofad500.1474

**Published:** 2023-11-27

**Authors:** Angela Berkhout, Julia Clark, Cheryl Jones, Brendan McMullan, Selina Lim, Daniel Yeoh, Philip Britton, Shirley Wong, Keith Grimwood, Daryl Cheng, Pamela Palasanthiran, Amanda Gwee, Jack Cross, Tran Nguyen, Emma Jeffs, Tony Walls, Michelle Mahony, Jennifer Yan, Clare Nourse

**Affiliations:** The Queensland Children's Hospital, Brisbane, Australia, Brisbane, Queensland, Australia; The Queensland Children's Hospital, Brisbane, Australia, Brisbane, Queensland, Australia; The University of Sydney, New South Wales, Australia, Sydney, New South Wales, Australia; Sydney Children's Hospital Randwick, New South Wales, Australia, Sydney, New South Wales, Australia; Perth Children's Hospital, Western Australia, Australia, Perth, Western Australia, Australia; Perth Children's Hospital, Western Australia, Australia, Perth, Western Australia, Australia; The Sydney Children's Hospital Network, Westmead, Sydney, New South Wales, Australia, Sydney, New South Wales, Australia; The Sydney Children’s Hospitals Network, Westmead, New South Wales, Australia, Sydney, New South Wales, Australia; The Gold Coast University Hospital, Queensland, Australia, The Gold Coast, Queensland, Australia; The Royal Children's Hospital, Melbourne, Victoria, Australia, Melbourne, Victoria, Australia; Sydney Children's Hospital, Randwick, Sydney, New South Wales, Australia, Sydney, New South Wales, Australia; The Royal Children's Hospital, Melbourne, Victoria, Parkville, Victoria, Australia; The Gold Coast University Hospital, The Gold Coast, Queensland, Australia, The Gold Coast, Queensland, Australia; The Sydney Children's Hospital, Randwick, Sydney, New South Wales, Australia, Sydney, New South Wales, Australia; University of Otago, Christchurch, New Zealand, Christchurch, Otago, New Zealand; University of Otago, Christchurch, Christchurch, Canterbury, New Zealand; The Royal Darwin Hospital, Northern Territory, Australia, Sydney, New South Wales, Australia; The Royal Darwin Hospital, Northern Territory, Australia, Sydney, New South Wales, Australia; The Queensland Children's Hospital, Brisbane, Australia, Brisbane, Queensland, Australia

## Abstract

**Background:**

Non-specific presentations of severe herpes simplex virus (HSV) infections and high risk of adverse outcomes have driven empiric acyclovir use. We therefore audited acyclovir prescribing in Australia and New Zealand for suspected HSV infection.

**Methods:**

All children (0-18 years) prescribed intravenous (IV) acyclovir for suspected HSV infection in eight paediatric hospitals in Australia and New Zealand between 1 January 2019 and 31 December 2019 were included. Clinical data were extracted from patient records.

**Results:**

IV acyclovir was prescribed for 1426 suspected cases, of whom 114 (8%) subsequently had proven HSV infection; 0.8% severe (9 encephalitis, 3 disseminated) and 7.2% (102) non-severe. Median age of the 1426 suspected cases was 4-months (IQR 0-49, range 0-223); 30% being neonates (<28 days), 17% aged 29 days-to-3 months and 53% aged >3-months. Suspected encephalitis (55%) and disseminated disease (29%) were the most common indications for prescribing acyclovir. 88% lacked risk factors and 90% had no potential identifiable source. 57% had CSF obtained, 25% and 13% had >1 surface swab and blood sent for HSV PCR testing respectively, whilst 20% had no HSV investigations. 34% were admitted to an intensive care unit. Median IV acyclovir duration was 1-day (IQR 1-2; range 0-81); 0.5% experienced nephrotoxicity and 2% had an extravasation injury. The median length-of-hospital stay was 4-days (IQR 2-9, range 0-307), 92% were well at discharge. Non-HSV infections (47%) and seizure disorders (15%) were the most common discharge diagnoses.

**Conclusion:**

This study suggests frequent unnecessary empiric acyclovir use, with 8% of children having proven HSV infection; minority severe (0.8%) and 20% of children not having any HSV investigations.

National algorithms identifying high-risk age groups and clinical features of neonatal HSV infection and HSV encephalitis are needed to better guide acyclovir use and limit unnecessary treatment.

**Disclosures:**

**All Authors**: No reported disclosures

